# Genomic analysis of *Elizabethkingia* species from aquatic environments: Evidence for potential clinical transmission

**DOI:** 10.1016/j.crmicr.2021.100083

**Published:** 2021-11-26

**Authors:** Sopheak Hem, Veronica M. Jarocki, Dave J. Baker, Ian G. Charles, Barbara Drigo, Sarah Aucote, Erica Donner, Delaney Burnard, Michelle J. Bauer, Patrick N.A. Harris, Ethan R. Wyrsch, Steven P. Djordjevic

**Affiliations:** a iThree Institute, University of Technology Sydney, Ultimo, NSW 2007, Australia; b Australian Centre for Genomic Epidemiological Microbiology, University of Technology Sydney, PO Box 123, Broadway, NSW 2007, Australia; c Quadram Institute Bioscience, Norwich, United Kingdom; d Norwich Medical School, Norwich Research Park, Colney Lane, Norwich NR4 7TJ, United Kingdom; e Future Industries Institute, University of South Australia, Adelaide, SA 5001, Australia; f University of Queensland Centre for Clinical Research, Royal Brisbane and Woman's Hospital, Building 71/918 Royal Brisbane and Women's Hospital Campus, Herston, QLD 4029, Australia

## Abstract

*Elizabethkingia* species are ubiquitous in aquatic environments, colonize water systems in healthcare settings and are emerging opportunistic pathogens with reports surfacing in 25 countries across six continents*. Elizabethkingia* infections are challenging to treat, and case fatality rates are high. Chromosomal *bla*_B_*, bla*_GOB_ and *bla*_CME_ genes encoding carbapenemases and cephalosporinases are unique to *Elizabethkingia spp*. and reports of concomitant resistance to aminoglycosides, fluoroquinolones and sulfamethoxazole-trimethoprim are known. Here, we characterized whole-genome sequences of 94 *Elizabethkingia* isolates carrying multiple wide-spectrum metallo-β-lactamase (*bla*_B_*and bla*_GOB_) and extended-spectrum serine‑β-lactamase (*bla*_CME_) genes from Australian aquatic environments and performed comparative phylogenomic analyses against national clinical and international strains. qPCR was performed to quantify the levels of *Elizabethkingia* species in the source environments. Antibiotic MIC testing revealed significant resistance to carbapenems and cephalosporins but susceptibility to fluoroquinolones, tetracyclines and trimethoprim-sulfamethoxazole. Phylogenetics show that three environmental *E. anophelis* isolates are closely related to *E. anophelis* from Australian clinical isolates (∼36 SNPs), and a new species, *E. umeracha* sp. novel*,* was discovered. Genomic signatures provide insight into potentially shared origins and a capacity to transfer mobile genetic elements with both national and international isolates.

## Highlights

• Identification of closely related (< 50 SNV) clinical and environmental aquatic *Elizabethkingia anophelis* isolates.
• Identification of a provisional novel species *Elizabethkingia umaracha*.
• Novel *bla*_GOB_ and *bla*_B_ carbapenemases and extended spectrum β-lactamase *bla*_CME_ alleles identified in *Elizabethkingia* spp.
• Analysis of the global phylogeny and pangenome of *Elizabethkingia* spp.
• Identification of novel ICE elements carrying uncharacterised genetic cargo in 67 / 94 (71.3%) of the aquatic environments *Elizabethkingia* spp.

## Graphical abstract

**Figure:**
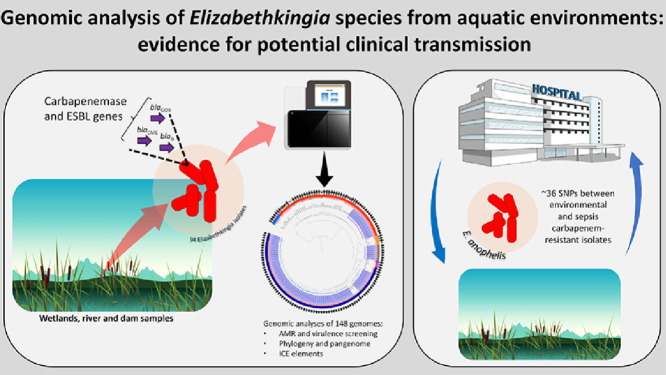

## 1 Introduction

The environment is a known reservoir for both opportunistic pathogens and antimicrobial resistant (AMR) bacteria (Ishii, 2019). It is important to investigate environmental microbial populations as prominent extended-spectrum β-lactamases (ESBLs), quinolone resistance genes and carbapenemases originated from marine and soil bacterium and have subsequently entered clinical isolates through horizontal gene transfer (HGT) (Poirel et al., 2005; Wyres and Holt, 2018), as well as identifying potential infection transmission pathways (Ishii, 2019).

*Elizabethkingia* species are aerobic, Gram-negative bacilli members of *Weeksellaceae* commonly found in the environment, particularly in soil and freshwater bodies, as well as insects and amphibians (Dworkin et al., 2006; García-López et al., 2019; Jean et al., 2014; Lei et al., 2019). There are currently six identified *Elizabethkingia* species, all of which have undergone various taxonomic and nomenclature makeovers (Lin et al., 2019a). *Elizabethkingia meningoseptica,* isolated in 1959, has been known as *Flavobacterium meningosepticum* and *Chryseobacterium meningosepticum* (King, 1959; Vandamme, 1994)*. Elizabethkingia miricola,* isolated from a Russian space station in (Li et al. (2003), has been known as *Chryseobacterium miricola* and *Elizabethkingia* genomospecies 2 (Holmes et al., 2013; Nicholson et al., 2018). *Elizabethkingia anophelis,* isolated in 2011, has been described as *Elizabethkingia endophytica* and *Elizabethkingia* genomospecies 1 (Doijad et al., 2016; Holmes et al., 2013; Kämpfer et al., 2015; Nicholson et al., 2018). In addition, three new species were redefined in 2017 – *Elizabethkingia bruuniana, Elizabethkingia ursingii* and *Elizabethkingia occulta -* the former previously been referred to as *Elizabethkingia* genomospecies 3 and the latter two were both grouped as *Elizabethkingia* genomospecies 4 (Holmes et al., 2013; Nicholson et al., 2018).

Interest in *Elizabethkingia* spp. is rising as they constitute difficult to treat emerging pathogens within hospitals and healthcare settings (Green et al., 2008; Lau et al., 2016; Teo et al., 2013). *Elizabethkingia* infections occur most often in newborns and immunocompromised patients, and the most common presentation is septicaemia (Sarma et al., 2011). However, reports of meningitis (caused by *E. meningoseptica* and *E. anophelis*), pneumonia, urinary tract infection, skin and soft tissue infections are also common (Lin et al., 2009; Venkatesh et al., 2018). Case fatality rates for *Elizabethkingia* spp. are high at ∼25.2% in all species (Seong et al., 2020) and higher in cases of septicaemia and meningitis at 54% for *E. meningoseptica* (Moore et al., 2016) and 28.4% for *E. anophelis* infections (Lin et al., 2018b). *Elizabethkingia* pathogenesis is largely unknown, though several virulence factor homologs have been reported, including capsule proteins, adhesins, iron uptake proteins and proteins contributing to biofilm formation (Chen et al., 2015; Janda and Lopez, 2017; Li et al., 2015; Liang et al., 2019).

Treatment of *Elizabethkingia* infections is complicated because most species are intrinsically resistant to clinically important antibiotics, including carbapenems and other β-lactams and aminoglycosides (Janda and Lopez, 2017; Li et al., 2015; Lin et al., 2019a). Resistance to fluoroquinolones and sulfamethoxazole-trimethoprim have also been observed (Lin et al., 2018a). Consistently, *Elizabethkingia* species genomes sequenced to date harbor multiple chromosomal antimicrobial resistance genes (ARGs), including genes *bla*_B_ and *bla*_GOB_, associated with resistance to carbapenems, and extended-spectrum β-lactamase gene *bla*_CME_, conferring resistance to all cephalosporins (González and Vila, 2012). For fluoroquinolones resistance, mutations within conserved regions of DNA gyrase subunit A (GyrA) have been observed, including Ser83Ile and Ser83Arg (Jian et al., 2018). ARGs have also been identified in *Elizabethkingia* integrative and conjugative elements (ICEs) (Xu et al., 2019), mobile genetic elements (MGEs) capable of integrating into a host genome and propagated during chromosomal replication and cell division (Wozniak and Waldor, 2010). Only two plasmids have been described and sequenced from two *Elizabethkingia* species: *E. anophelis* strain F3201 (Xu et al., 2019) and *E. miricola* strain EM_CHUV (Opota et al., 2017).

Cases of *Elizabethkingia* infections have been increasing over the past few decades, with reports surfacing in 25 countries across six continents (Breurec et al., 2016; Burnard et al., 2020; Hsu et al., 2011; Lau et al., 2015; Lin et al., 2019b; Perrin et al., 2017; Reed et al., 2020; Teo et al., 2013). *Elizabethkingia* mode of transmission remains unclear, but exposure to contaminated environments, especially waterbodies, medical devices, hemodialysis and mechanical ventilation equipment, hospital fomites, water faucets and healthcare worker hands, have all been implicated (González and Vila, 2012; Lin et al., 2018a). In addition, infections caused by *E. anophelis* have been linked to transmission events associated with mosquitoes in the Central African Republic (Frank et al., 2013); however, this hypothesis is contentious due to a report of vertical transmission from mother to infant (Lau et al., 2015).

*Elizabethkingia* species are multidrug-resistant emerging pathogens with high case-fatality rates. To date, most studies on *Elizabethkingia* have characterized clinical isolates (Eriksen et al., 2017). However, given that waterbodies are reservoirs and implicated in *Elizabethkingia* transmission pathways, this study provides a genomic analysis of whole-genome sequences (WGS) derived from 94 *Elizabethkingia* isolates originating from aquatic environments in South Australia, as well as characterizing and comparing their genomic and antimicrobial resistance profiles to other publicly available *Elizabethkingia* environmental and clinical strains.

## 2 Materials and methods

### 2.1 Sample collection and bacterial isolation

Water samples (∼10 L) were collected monthly in triplicate from July 2018 to July 2019 in South Australia. The four locations represented two sources: (i) stagnant water (site A) and (ii) inland wetlands recharged by seasonal rainfall/runoff inflows (site B and C) or by a river (site D). Site A was a small rural reservoir created by damming a natural rainwater catchment area. It was fenced and not accessible to or impacted by livestock but was regularly visited by birds and particularly by ducks. Site B (wetland) was a recreation reserve covering an area of 19.4 hectares. Site C (wetland) covered 172 hectares and had a maximum capacity of 1200 megaliters. The distance between the two wetland sites was ∼10 km. Site D was a river 2508 km in length, and the area sampled was flowing water near a wetland that covers a total of 42 hectares. Site D was used for recreational purpose only. At all sites, surface water samples were collected by dipping three sterile 10 L collection tanks below the surface. All samples were stored on ice directly after collection and processed within 2–3 h.

### 2.2 Isolation of carbapenem-resistant *Elizabethkingia* spp

Samples were processed on the day of collection. First, water samples were serially diluted and 500 μl from 2 to 3 consecutive 10-fold serial dilutions were plated in triplicate on Oxoid *Brilliance*™ CRE Agar plates (Thermo Fisher Scientific Australia, Adelaide, SA). Cultures were incubated at 25 °C, 37 °C and 44 °C for 24 h. Next, using pre-sterilized toothpicks, single colonies growing on CRE Agar were plated on Plate Counting Agar (PCA; Thermo Fisher Scientific). PCA cultures were incubated at 37 °C for 18–24 h, or until sufficient bacterial growth had occurred. A total of 667 bacteria were isolated and identified with Matrix-Assisted Laser Desorption Ionization-Time of Flight Mass Spectrometry (MALDI-TOF MS) (Bruker Daltonics) and preserved in glycerol stocks (40% v/v) at −80 °C.

### 2.3 MALDI-TOF MS species identification

Fresh bacterial single isolates (<24 h old) were resuspended in 1 ml 70% ethanol, vortexed for 1 min, and centrifuged at 13,000 rpm for 2 min. The supernatant was removed, and the pellet re-dissolved with 5 µl of 70% formic acid (Baker; 90% stock) and 5 µl acetonitrile (CAN, LC-MS Grade, Merck). After 2 min of centrifugation at 13,000 rpm, 1 µl of supernatant was spotted onto the target plate and left to dry. The sample was overlaid with α-cyano-4-hydroxycinnamic acid (HCCA) (1 μl) matrix (10 mg/ml^−1^) and allowed to crystallize at room temperature. One μl of Bacterial test standard (Bruker Daltonics) in 50% (v/v) ACN containing 2.5% (v/v) trifluoroacetic acid (LC-MS Grade; Thermo Fisher Scientific) was spotted, left to dry and overlaid with HCCA for calibration. MALDI-TOF MS analysis was acquired on an autoflexTM speed MALDI-TOF/TOF mass spectrometer (Bruker Daltonics) operated in linear positive mode under MALDI Biotyper 3.0 Real-time Classification (version 3.1, Bruker Daltonics) and FlexControl (version 3.4, Bruker Daltonics) software. Spectra were acquired in the mass range of 2000 to 20,000 Da with variable laser power, and a total of 1200 sum spectra were collected in 40 shot steps. The sample spectra were identified against an MSP database library (5989 MSP entries). Identification scores of 2.300–3.000 indicate highly probable species identification, scores of 2.000–2.299 indicate secure genus identification and probable species identification, scores of 1.700–1.999 indicate probable genus identification, and a score of ≤ 1.699 indicates that the identification is not reliable.

### 2.4 DNA extraction

Water samples were concentrated by vacuum filtration through a 0.2 μm nitrocellulose filter (Millipore) then stored at −80 °C until DNA extraction. Total genomic DNA from each 0.2 μm filter was extracted using the DNeasy PowerWater kit (Qiagen) according to the manufacturer's instructions. DNA from MALDI-TOF MS identified colonies with scores 2.000–3.000 were extracted using the DNeasy Blood & Tissue Kit (Qiagen) according to the manufacturer's instructions. Nucleic acid quality (i.e., 260/280 ratio) was measured with Nanodrop 1000 spectrophotometer (Thermo Fisher Scientific). DNA concentrations for all samples were measured by fluorometric quantitation using a Qubit instrument and High Sensitivity dsDNA HS Assay kit (Thermo Fisher Scientific), and purified DNA extracts were stored at −20 °C until used.

### 2.5 Absolute quantification of *Elizabethkingia* spp., *E. anophelis* and *E. meningoseptica*

Standard curves to determine the absolute quantity, efficiency, linear range, and reproducibility of *Elizabethkingia* spp., *E. anophelis* and *E. meningoseptica* assays were prepared using the American Type Culture Collection (ATCC) strain *E. meningoseptica* ATCC 13,253 and the clinical isolate identified by MALDI-TOF and whole genome sequencing *E. anophelis* DSM 23,781. The ATCC strain and the clinical isolate were purified using the QIAamp DNA Mini and Blood Mini kit according to the manufacturer's protocol for isolation of genomic DNA from bacterial plate cultures (page 56, Handbook 05/2016, Qiagen, Sydney, NSW). The DNA concentration and quality were determined using a Qubit 2.0 fluorometer (Life Technologies). Standards were prepared by serial diluting the DNAs and by calculating *E. anophelis* and *E. meningoseptica* copy number with the following equation:

*Elizabethkingia* copy number = (concentration of template in ng × *NL*) / (*n* × 10^9^ × *660*) where *NL* is the Avogadro constant (6.02 × 10^23^), *n* is the genome length of the standard in base pairs or nucleotides and *660* is the average molecular weight of double-stranded DNA.

Digital droplet PCR (ddPCR) was used to quantify the copy numbers of the standard's serial dilutions. ddPCR was performed using QX200™ ddPCR™ Supermix for Probes (No dUTP, Biorad, Australia) and a QX200™ Droplet Digital™ PCR System with automated droplet generation (Bio-Rad, Pleasanton, CA, USA). All ddPCR amplifications were conducted in 20 μL reaction mixtures containing 10 μL of Probe Supermix, 1 μL of each individual primer (100 nM), 2 μL of template DNA, and 6 μL of ultrapure PCR-grade water. The ddPCR amplification conditions were as follows: 25 °C for 3 min, 95 °C for 10 min, 40 cycles at 94 °C for 30 s and 60 °C for 1 min, 98 °C for 10 min and 4 °C for hold.

All qPCR analyses were carried out in duplicate on a LightCycler® 480 Instrument II (Roche Life Science) with positive, negative, and non-template controls included. Individual real-time qPCR assays were used to quantify *E. anophelis, E. meningoseptica* and *Elizabethkingia* spp. genome copies using a multiplex probe assay with the primers and probes described in Table 1.

**Table 1:** Gene targets, primers and probes used in this study.

Gene target	Target organism	Primer/Probe ID	Fluorophore/Quencher	Final reaction conc (μM)	Product size (bp)	Primer sequence (5′−3′)	Reference
*secY*	*Elizabethkingia* spp.	SECYF1_4		0.01		GTTTTTACGTTCACGCTCATCTTGGT	Kelly et al. (2019)
		SECY R2		0.07	146	AGTAAGCCTAAAAGCCCAGAAG	
		SECYP2_5	FAM/BHQ1	0.05		TTGCAAGTATACAGAACCAAGGAGGAAGCAAG	
*pheT*	*E. meningoseptica*	TIGR472_F7		0.1		TTTAAACTGGATGTGGAAGATGCTGAT	Kelly et al. (2019)
		TIGR472_R1_2		0.05	90	CCACTCTGGGGACTCTTCTACCTGT	
		TIGR472_P3	Quasar 670/BHQ3	0.05		GCGTTATCTGGGAGCTGTAATTGAAGG	
*lepA*	*E. anophelis*	TIGR1393F22		0.07		CATGTGAAGGGGCGCTACTTATTGT	Kelly et al. (2019)
		T1393R3WT		0.1	142	TCAGGGTTTGCAGAAGGAAGGTC	
		TIGR1393P1	CalRed 610/BHQ1	0.02		ACCTGGCTTTGGAAAATGACCTTACC	

Amplification was done in 25 µl reaction volumes consisting of 10 µl of the LightCycler® 480 SYBR Green I Master (Roche Life Science), 5 µl of DNAse/RNAse free water (Roche Life Science), 5 µl of the primer-probe mixture, and 5 µl of template DNA within the concentration range of 40 to 50 ng/µl. The cycling conditions were as follows: initial denaturation at 95 °C for 3 min, followed by 40 cycles of 95 °C for 10 s and 64 °C for 30 s (Kelly et al., 2019). Fluorescence data were acquired at the end of the annealing step of each cycle. All mixes were made using a Biomek Automated Liquid Handler (Beckman Coulter) to avoid pipetting errors. The efficiency of the different real-time PCRs ranged from 97 to 100%. Secondary structures were not encountered in any of the runs. The threshold of each single run was placed above any baseline activity and within the exponential increase phase. The cycle thresholds (C_T_) were determined by a mathematical analysis of the resulting curve using the software manufactured by Roche Life Science. The C_T_ values of the non-template controls were always 40 or above, indicating no amplification. Dissociation curves were determined for qPCR products to confirm product integrity and the absence of PCR inhibitors. Among the different qPCR coefficients, attention was given to the R coefficient, which was used to analyze the standard curves obtained by linear regression analysis. Most of the samples, and all standards, were assessed with a minimum of two runs to confirm the reproducibility of the quantification.

Real-Time PCR datasets were analyzed using analysis of variance (ANOVA). To evaluate the absolute abundance of gene copy numbers in water samples, F-tests were used to compare variance. Normality was tested with a Shapiro-Wilks test and by inspection of residuals, and variance homogeneity by Levene's test. When data failed to satisfy one of these tests, an appropriate transformation was applied (log or square-root transformation). Tukey's honestly significant difference (HSD) method and the modified version for unequal sample size (Unequal N HSD) were used for post hoc comparisons with a 0.05 grouping baseline. Graphs were drawn using GraphPad Prism version 9 (GraphPad Software Inc.).

### 2.6 Whole-genome sequencing

Whole-genome sequencing was performed as described previously (Foster-Nyarko et al., 2020). Briefly, WGS was performed on the Illumina NextSeq 500 platform using a modified Nextera low input tagmentation approach. Genomic DNA was normalized to 0.5 ng µl−1 with 10 mM Tris–HCl before the library preparation. The pooled library was run at a final concentration of 1.8 pM on a mid-output flow cell following Illumina recommended denaturation and loading parameters. Data was uploaded to Basespace (www.basespace.illumina.com), where the raw data was converted to FASTQ files for each sample.

### 2.7 Phylogenetic analysis

Maximum-likelihood phylogenetic trees were constructed using PhyloSift (Darling et al., 2014), and single nucleotide polymorphism (SNP)-based phylogenetic trees were made using Snplord (github.com/maxlcummins/pipelord/tree/master/snplord), an automated snakemake (Köster and Rahmann, 2012) pipeline that utilizes snippy (github.com/tseemann/snippy), Gubbins (Croucher et al., 2015) and SNP-sites (github.com/sanger-pathogens/snp-sites). All trees were resolved using FastTree2 (Price et al., 2010) and visualized using the Interactive Tree Of Life (iTOL) software v4 (Letunic and Bork, 2019). In addition to the 94 *Elizabethkingia* draft genomes presented in this study, 54 *Elizabethkingia* genomes sourced from Genbank (Leray et al., 2019) were included in the phylogenetic analyses. The *Elizabethkingia* pangenome was calculated using Roary v3.11.2 (Page et al., 2015) and visualized using Phandango v1.3.0 (Hadfield et al., 2018). A pangenome wide gene association study on novel *Elizabethkingia* spp. isolates was performed using Scoary (Brynildsrud et al., 2016). A pairwise genome distance matrix was generated using Mash (Ondov et al., 2016) and used to create a classical (metric) multidimensional scaling (MDS) plot using R Studio v4.0.2 and the gglot2 v3.3.0 package. MDS plots for virulence-associated genes and ARGs were also created in R, using gene presence/absence matrices (1 = present; 0 = absent).

### 2.8 Genotyping

*In silico* species identification was performed using SpeciesFinder 2.0 (Larsen et al., 2014) and Kraken2 (Wood et al., 2019). To determine novel *Elizabethkingia* species, pairwise genome comparisons were performed using both the average nucleotide identity BLASTn (ANIb) and ANI MUMer (ANIm) algorithms available on the JSpecies web server (Richter et al., 2016) using a 95% cut-off value for species delimitation (Goris et al., 2007). Predicted DNA-DNA hybridization (DDH) results were ascertained using the Genome-to-Genome Distance Calculator (GGDC) tool (Meier-Kolthoff et al., 2013) with a 70% cut-off value for species delimitation using the recommended Formula 2. Complete 16S rRNA and *rpoB* sequences were aligned using Clustal Omega and 99.5% (Sievers and Higgins, 2018) and 97.7% (Adékambi et al., 2009) similarity cut-off values were used, respectively. Virulence-associated genes, ARGs and plasmid replicons were screened for using Abricate (github.com/tseemann/abricate) in conjunction with the following databases: VFDB (Chen et al., 2005), CARD (Alcock et al., 2019), NCBI AMR FinderPlus (Feldgarden et al., 2019) and PlasmidFinder (Carattoli et al., 2014). Virulence factors were also screened using the VFDB Set A of experimentally determined virulence factors and BLASTp with > 40% amino acid sequence identity and E^−10^ cut-off values.

### 2.9 Genome annotation

Draft genomes were annotated using Prokka v1.14.6 (Seemann, 2014) and managed using SnapGene v4.1.9 (snapgene.com). The RAST annotation pipeline (Brettin et al., 2015) was also utilized on eight genomes representative of each clade to cross check annotations. Putative genomic islands (GIs) and ICEs were identified by Islandviewer 4 (Bertelli et al., 2017) using the following reference genomes: *E. anophelis* strain CSID_3,015,183,681 (CP015068.2), *E. anophelis* strain F3543 (CP014340.1), *E. miricola* strain EM798–26 (CP023746.1) and *E. genomospecies 4* strain G4123 (CP016377.1). BLASTn was utilized to determine whether putative GIs, ICE and AMR regions identified in this study had been previously deposited into NCBI. Aliview software v3.0 (GPLv3) (Larsson, 2014) was used to view the sequence alignment of AMR genes.

### 2.10 Minimum inhibitory concentration (MIC) testing

Representative isolates from each *Elizabethkingia* clade and isolates harbouring unique combinations of *bla*_B_ *and bla*_GOB_ genes were selected for MIC testing (*n* = 10) against 38 clinically relevant antimicrobials as described previously (Burnard et al., 2020). Antibiotic testing plates were hand prepared, inoculated and incubated in accordance to AS ISO 20,776.1–2017. Quality Control of antibiotic and testing isolates was in accordance to Clinical and Laboratory Standards Institute (CLSI) M100 ED31:2021; plate reading in accordance to the European Committee on Antimicrobial Susceptibility Testing (EUCAST) reading guide v 3.0 2021. Both the guidelines of the EUCAST pharmacokinetic-pharmacodynamic (PK-PD) “non-species” breakpoints (Kahlmeter et al., 2006) and the non-*Enterobacteriaceae* breakpoints of the CLSI (Jorgensen et al., 2007) were used in the AMR phenotypic analysis.

## 3 Results

In this study, 94 *Elizabethkingia* isolates were collected from aquatic environments in South Australia from 2018 to 2019. Strains sourced from wetlands (site B & C) constituted the majority [*n* = 70 (*B* = 50, C = 20); 75%], followed by dam (site A, *n* = 22; 23%) and then river (site D, *n* = 2; 2%) samples. Associated metadata on all isolates used, including 54 sourced from outside this collection used in phylogenetic and gene screening analyses, is available in Supplementary Data 1.

### 3.1 Genome assembly

Draft genomes were assembled using shovill v1.0.4. Genome size ranged from 4,039,979 bp to 4,660,922 bp, with an average size of 4,459,168 bp. The number of contigs per genome ranged from 25 to 160, with a mean of 55. Read depth ranged from 23.26 to 80.63, with a mean of 38.79. Full assembly statistics can be viewed in Supplementary Data 2.

### 3.2 Absolute quantification of *Elizabethkingia* spp., E. *anophelis* and E. *meningoseptic*a

**Fig. 1:**
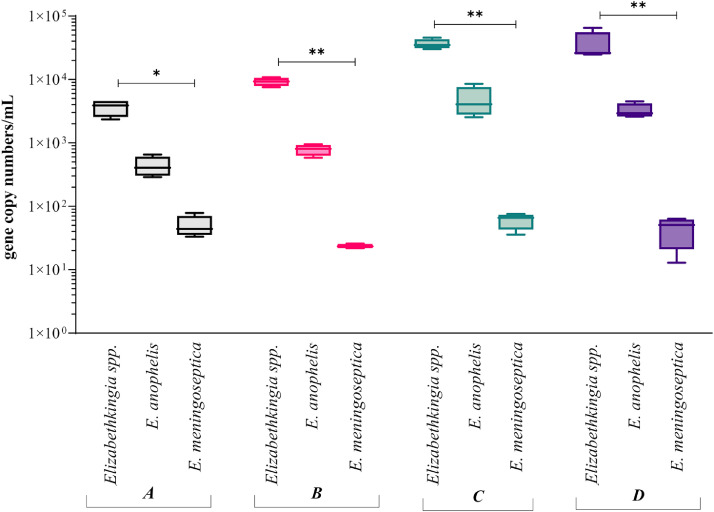
*Elizabethkingia* spp., *E. anophelis* and *E. meningoseptica* average absolute abundance determined by qPCR analysis of total DNA extracted from filtered waters (site A, B, C and D). Data are expressed as log10 genes copies per mL, samples (*n* = 32). Asterisks denote: * = *P* < 0.05; ** = *P* < 0.01.

Quantitative data targeting a generic *Elizabethkingia* spp. gene marker and *E. anophelis* and *E. meningoseptica* markers were used to estimate the absolute abundance of each in samples from the four aquatic sites. The absolute abundance of *Elizabethkingia* spp. in the dam sample was on average 7.6 × 10^3^ gene copies/mL, representing 1.36×10^−6^ of the total bacterial community (16S rRNA qPCR-based). In the wetland samples, *Elizabethkingia* spp. ranged from 3.5 × 10^4^ genes/mL to 4.6 × 10^4^ genes/mL, representing 6.25×10^−6^ to 8.21×10^−6^ of the total bacterial community (Fig. 1). In each case, the absolute abundance of *E. anophelis* was a factor of ten lower than the total *Elizabethkingia* spp. absolute abundance, indicating that it is not the dominant species within the aquatic environments. *E. meningoseptica* were detected and gene copies/mL were ranging from 23 (site A; dam) to 50 copies/mL (site B, C and D; wetlands) on average.

### 3.3 Identification of *Elizabethkingia* species

The speciation of *Elizabethkingia* isolates varied considerably between the typing techniques implemented (Table 2). Of the 94 isolates sourced from Australian aquatic environments, the most prominent species identified by MALDI-TOF MS was *E. miricola* (*n* = 54; 57%), by Kraken2 was *E. anophelis* (*n* = 93; 99%), and according to SpeciesFinder 2.0 E. genomospecies 4 was most prevalent (*n* = 77; 82%). Our phylogenetic characterization (detailed below) classified 71 isolates as *E. miricola* (76%), 16 isolates as *E. anophelis* (17%) and seven isolates as a potentially novel species (7%).

**Table 2:** Aquatic environmental *Elizabethkingia* species (*n* = 94) identified by MALDI-TOF MS, Kraken2, Species Finder, and phylogenetic analysis.

Species Identification	MALDI-TOF MS	Kraken2	SpeciesFinder 2.0	Phylogenetic characterization
*E. anophelis*	0	93 (99%)	16 (17%)	16 (17%)
*E. meningoseptica*	11 (12%)	0	0	0
*E. miricola*	54 (57%)	0	1 (1%)	71 (76%)
E. ursingii*	0	1 (1%)	77 (82%)	0
*Elizabethkingia* spp.	16 (17%)	0	0	7 (7%)
Non-Reliable Identification	13 (14%)	0	0	0

⁎ Also known as *E*. genomospecies 4.

### 3.4 Phylogenetic analysis

**Fig. 2:**
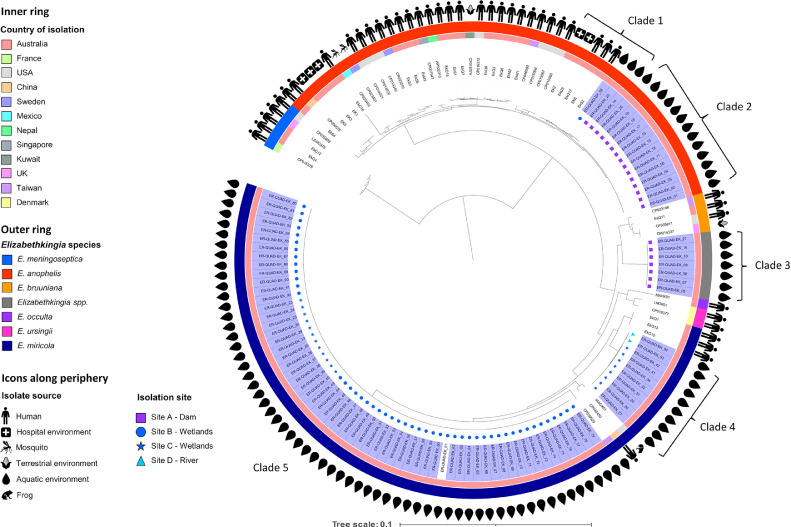
*Eizabethkingia* phylogeny. Mid-point rooted maximum likelihood phylogenic tree and geographic data of *Elizabethkingia* species using Phylosift. Samples from this collection are coloured in purple.

A phylogenetic tree comprised of 148 *Elizabethkingia* isolates was constructed using Phylosift (Fig. 2) with 94 isolates from the Australian aquatic environments (this collection), 27 isolates from Australian clinical samples and Australian hospital environments, and 27 international strains available from Genbank. Where metadata was available, *Elizabethkingia* isolates were derived from the environment (*n* = 102), humans (*n* = 42), *Anopheles gambiae* (*n* = 2), and one isolate each from *Zea mays* (corn) and a frog. The distribution of species in the phylogenetic tree were *E. anophelis* (*n* = 52), *E. meningoseptica* (*n* = 5), *E. miricola* (*n* = 78), *E. bruuniana* (*n* = 3), *E. ursingii* (*n* = 2), *E. occulta* (*n* = 1) and a novel clade of *Elizabethkingia* spp. (*n* = 7). The seven species of *Elizabethkingia* were clearly separated from each other, with *E. meningoseptica* appearing the most distant from the other species.

The *Elizabethkingia* isolates from our aquatic environment study formed five clades that branched alongside international and clinical isolates. Clade 1 contained three *E. anophelis* from the aquatic environmental study (ER-QUAD-EK_54, QUAD-EK_14 and QUAD-EK_22) that were closely related to Australian clinical isolates (EkS2, EkQ5 and EkQ17) with an average of 36 SNV (Single nucleotide variants) across 87% of the core genome (EkS2 as reference) and hospital environment isolates EK2 and EK6 with an average of 42 SNV. The two *E. anophelis* isolates from dam samples, QUAD-EK_14 and QUAD-EK_22, were separated by 2 SNV, while ER-QUAD-EK_54 from the Australian wetland samples differed by an average of 33 SNV from dam isolates. Clade 2 consists of 13 clonal *E. anophelis* from our study that differ by an average of 2 SNV between each other and 807 SNV from our *E. anophelis* isolates situated in Clade 1 (pairwise SNP matrices for *E. anophelis* isolates provided in Supplementary Data 4). Clade 3 appears as a novel clade, represented by seven isolates (ER-QUAD-EK_21, QUAD-EK_08, QUAD-EK_09, QUAD-EK_10, QUAD-EK_16, QUAD-EK_07, and QUAD-EK_05) isolated from an Australian dam. Isolates within this apparently novel clade of the *Elizabethkingia* were most closely related to *E. bruuniana* but appeared genetically distinct in a progressiveMauve analysis (Supplementary Data 3) and differed by an average of 124,216 SNPs to E. bruuniana isolate EkQ11. Clade 4 constitutes ten *E. miricola* isolates with an average of 66 SNV between each other (range 0 – 197 SNPs) across 83% of the core genome (EkQ1 as reference). These isolates branch alongside three *E. miricola* isolates from Australian clinical samples (EkQ10, EkQ13 and EkQ1) however, the SNV between these two branches is ∼21,539. Clade 5 represents a group of 61 clonal *E. miricola* isolates (average 7 SNV) from Australian wetlands, with the closest relative strain CP03929, from a water sample from Taiwan, at ∼21,629 SNPs difference. Pairwise SNP matrices for *E. miricola* isolates provided in Supplementary Data 4.

### 3.5 Identification of proposed new species *Elizabethkingia umeracha* sp. nov

The seven isolates in clade 3, with an average 124,216 SNV to *E. bruuniana* isolate EkQ11, were investigated to determine whether they constituted a closely related, yet distinct species to *E. bruuniana*. For this purpose, 16S rRNA and *rpoB* sequence identities as well as ANIb, ANIm and GGDC values (the latter mimicking DDH values) were calculated (averages presented in Table 3; full analysis in Supplementary Data 5). Except for a single ER-QUAD-EK_05 16S rRNA sequence identity result (99.7%), all other values placed these seven isolates as representing a novel *Elizabethkingia* species. We therefore propose that these seven isolates constitute a provisional novel species and propose the name *Elizabethkingia umeracha*; Umeracha meaning “fine waterhole” in the Peramangk language. We respectfully acknowledge the Peramangk people as the traditional owners and custodians of the waters and lands of the Adelaide Hills where these isolates originated.

**Table 3:** *E. umeracha* sp. nov. is a separate species from *E. bruuniana*, as evidenced by ANIb, ANIm, GGDC, and 16S rRNA and *rpoB* sequence identity.

	Average values for seven *E. umeracha* sp. nov. isolates				
	ANIb(>95% cutoff)	ANIm(>95% cutoff)	Predicted DDH (>70% cutoff)	16S rRNA (>99.5% cutoff)	rpoB(>97.7% cutoff)
*E. bruuniana* str. ATCC 33,958 (CP035811)	76.70 ± 0.73 SD	78.18 ± 0.57 SD	49.23 ± 0.04 SD	99.28 ± 0.17 SD	97.59 ± 0 SD
*E. bruuniana* str. G0146 (CP014337)	76.41 ± 0.71 SD	77.90 ± 0.65 SD	45.18 ± 0.07 SD	99.28 ± 0.17 SD	97.59 ± 0 SD
*E. bruuniana* str. EkQ11 (SRS5502615)	76.57 ± 0.70 SD	78.27 ± 0.65 SD	49.17 ± 0.43 SD	99.41 ± 0.18 SD	97.62 ± 0 SD

### 3.6 Pangenome analysis

**Fig. 3:**
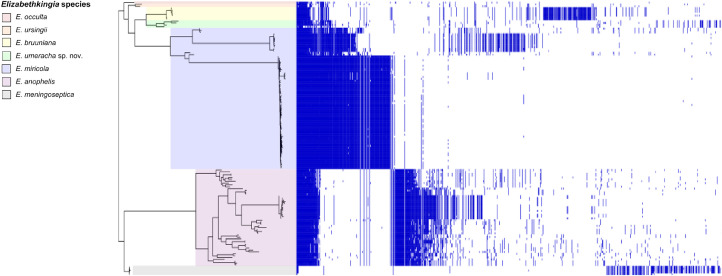
*Elizabethkingia* pangenome. Pangenome analysis of 148 *Elizabethkingia* species from the Australian environment and clinical isolates alongside international strains.

A pangenome analysis of all available *Elizabethkingia* genomes (*n* = 148) demonstrated high genetic diversity (Fig. 3). The *Elizabethkingia* spp. pangenome consisted of 28,240 genes, with a core genome of only 76 genes and an accessory genome of 28,164 genes (443 soft-core, 6057 shell and 21,664 cloud genes).

**Fig. 4:**
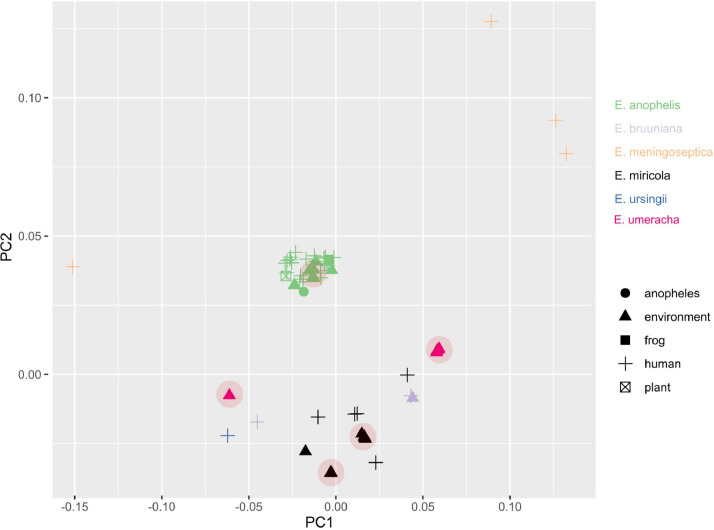
*Elizabethkingia* pairwise genome distances. MDS illustrating pairwise genome distances calculated using Mash. Colored by species, shapes represent isolate source. Red areas are isolates from this collection.

A pairwise genome distance MDS plot of *Elizabethkingia* genomes (Fig. 4) demonstrated tight clustering of all *E. anophelis* isolates, while *E. meningoseptica* isolates were the most distinct, both regarding other species and also between the *E. meningoseptica* isolates. The remaining *Elizabethkingia* species formed a more diffuse cluster with no clear distinction between human and environmental isolates and with *E. umeracha* sp. nov. isolates situated at the peripheries (Fig. 4, pink triangles).

The gene presence/absence matrix generated by Roary (Supplementary Data 6) was fed into Scoary to calculate any differentiating genes present in *E. umeracha* sp. nov. isolates. A total of 1886 genes were only identified in these seven isolates (100% specificity, 100% sensitivity; Supplementary Data 7). More than half of these genes (*n* = 1110; 58.8%) encode hypothetical proteins however of the remaining genes, 537 were fed into STRING which identified several functional enrichments, the highest scoring being tryptophan biosynthesis (1.04 strength) and molybdenum cofactor biosynthesis (1.04 strength) (full analysis available in Supplementary Data 8).

### 3.7 Virulent gene identification

**Fig. 5:**
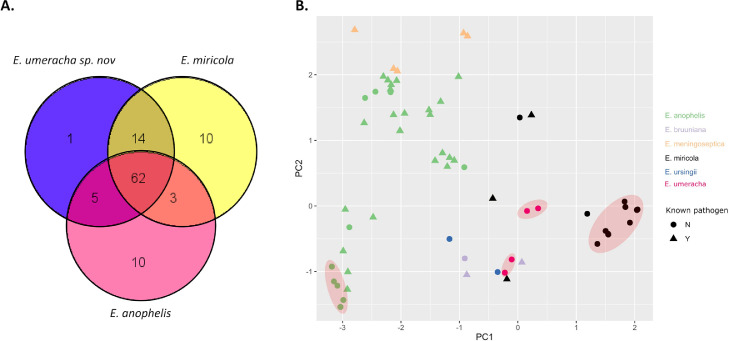
Virulence factors of *Elizabethkingia* species. (A) Venn diagram of distribution of putative virulence factors across the three *Elizabethkingia* species identified in this collection. (B) MDS analysis of putative virulence factors identified in 148 *Elizabethkingia* isolates. Colored by species. Triangles = known pathogen; circles = ability to cause disease unknown. Red areas = isolates from this collection.

Using the VFDB, a total of 107 putative virulence factors were identified in this collection with 62 (56%) of these being shared across the three identified species (Fig. 5A). However, several virulence factors were species specific. In *E. miricola*, unique virulence factors included homologs of adhesin/invasin Cj1136 (found in all *E. miricola* isolates, *n* = 71; 100%, notably absent in *E. miricola* isolates outside this collection) (Javed et al., 2012), capsule protein Cps41 (*n* = 71; 100%) (Auger et al., 2018), adenylate cyclase CyaB (*n* = 8; 11%) (Ante et al., 2021), ABC-transporter HlyB (*n* = 8; 11%) (Benabdelhak et al., 2003), toxins RtxB (*n* = 11; 15%), RtxE (*n* = 10; 14%) (Ramamurthy et al., 2020) and SmcL (*n* = 71, 100%) (González-Zorn et al., 2000), immune evasion protein GtrB (*n* = 8; 11%) (Xiao et al., 2021), intracellular growth protein PrsA2 (*n* = 1) (Alonzo and Freitag, 2010) and iron uptake protein YbtP (*n* = 71; 100%) (Fetherston et al., 1999). The virulence factors only identified in *E. anophelis* isolates were homologs of capsule proteins WbaP (*n* = 1) (Ernst et al., 2020), Cj1440c (*n* = 10; 63%, only found in E. anophelis from this collection) (Karlyshev et al., 2005), FTT_0790 (*n* = 1), FTT_0797 (*n* = 16; 100%), and FTT_0798 (*n* = 3; 19%) (Rowe and Huntley, 2015), lipopolysaccharide proteins BplB, BplG (Novikov et al., 2019), and KfoC (Lapp et al., 2021) (all *n* = 16; 100%), immune evasion protein OmpA (Vila-Farrés et al., 2017) (*n* = 16; 100%), and stress protein MucD (Yorgey et al., 2001) (*n* = 1). Only one unique virulence factor homolog was identified in *E. umeracha* sp. nov. – capsular protein NeuB (Feng et al., 2012) - however it was only identified in two isolates (29%).

An MDS analysis on putative virulence factors identified in this collection as well as genomes sourced outside of this collection (Fig. 5B; virulence factor BLAST results and heatmaps in Supplementary Data 9) demonstrated that our *E. anophelis* isolates clustered with a subset of known pathogenic *E. anophelis* isolates while our *E. miricola* isolates formed a cluster of their own. *E. umeracha* sp. nov. isolates formed two separate clusters, one standalone, the other amongst pathogenic *E. bruuniana* and *E. miricola* isolates and an *E. usingii* isolate.

### 3.8 Elizabethkingia AMR

#### 3.8.1 Beta-lactamase resistance genes

All 94 *Elizabethkingia* isolates from this Australian aquatic collection carried *bla*_B_ (subclass B1) and *bla*_GOB_ (subclass B3) genes encoding resistance to carbapenems and a *bla*_CME_ gene encoding resistance to cephalosporins.

**Fig. 6:**
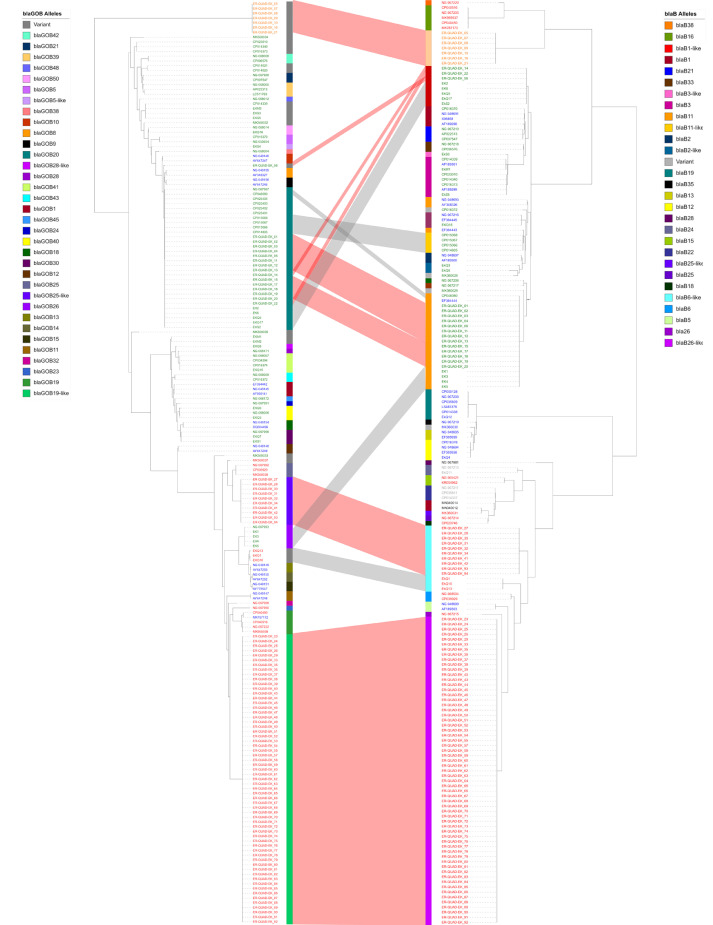
*Elizabethkiniga bla*_GOB_ and *bla*_B_ alleles. Phylogenetic trees of all *Elizabethkingia bla*_B_ and *bla*_GOB_ alleles. Left side is the tree of *bla_GOB_* alleles and right side is the tree of *bla_B_* alleles. Labels are colored in red for *E. miricola*, green for *E. anophelis*, blue for *E. meningoceptica* and orange for *E. umeracha* sp. nov. Connecting space between the trees links sequences from the same isolate. Available allele numbers are presented as colored strips.

MUSCLE alignments with all available reference sequences of *bla_B_* and *bla_GOB_* were generated to compare species and allele distributions (Fig. 6). All *Elizabethkingia* species in our analysis carried *bla*_GOB_, with an interesting distribution of several distinct signature deletions of 2–4 amino acids within the different alleles (Supplementary Data 10). However, none of these deletions are expected to alter gene reading frame given they appear in multiples of three nucleotides. For the *bla*_B_ gene distribution (Fig. 6; right side tree), we saw four to five primary clades in the tree structure which were generally grouped by species.

Regarding metallo-β-lactamase allele combinations, one *E. anophelis* isolate (ER-QUAD-EK_56) carried a novel *bla*_GOB_ variant and the remaining 15 *E. anophelis* isolates from this study carried *bla*_GOB-20_. Of these 15 isolates, 13 carried *bla*_B-11_. Interestingly, the remaining 3 *E. anophelis* isolates (ER-QUAD-EK-14, −22, and −56), carried a *bla*_B-1-like_ gene also shared by three Australian clinical isolates (EkS2, EkQ5, EkQ17) and two Australian hospital environment isolates (EK2 and EK6). Together these eight isolates formed the closely related (∼36 SNPs to clinical isolates; ∼42 SNPs to hospital environment isolates) clade 1 depicted in Fig. 2.

The ten *E. miricola* isolates of clade 4 uniquely carried *bla*_GOB-25-like_ genes and all carried *bla*_B6-like_ genes. These *bla*_B6-like_ genes were also identified in three Australian clinical *E. miricola* isolates (EkQ1, EkQ10 and EkQ13). The remaining 61 *E. miricola* isolates from this study uniquely carried *bla*_GOB19-like_ and *bla*_B26-like_ genes. Notably, *E. umaracha* sp. nov. isolates carried novel alleles of both metallo-β-lactamase genes.

Chromosomal extended-spectrum β-lactamase bla_CME_ has two types known: *bla*_CME-1_ and *bla*_CME-2._ *bla*_CME-1_ appeared to be the closest allele related to the Australian aquatic environmental *E. anophelis* (ER-QUARD-EK_14, 22, 56) from wetland and dam samples. *bla*_CME-2_ was present at very high levels of variation from the aquatic environment isolates. Interestingly, the two distinct *E. miricola* clades appear to possess each a novel *bla*_CME_ allele, and a third novel allele appears in the *E. umeracha* sp. nov. (Supplementary Data 11).

#### 3.8.2 Other ARGs

No other ARGs were detected in any of the Australian aquatic environment isolates. We also searched for the known mutations in *gyrA* (Ser83Ile or Ser83Arg) that encode resistance to ciprofloxacin and levofloxacin (fluoroquinolone), however none were detected.

#### 3.8.3 AMR phenotypic analysis: MIC testing

Ten representative isolates of *E. anophelis, E. miricola* and *E. umeracha* sp. nov., harboring unique combinations of *bla*_B_, *bla*_GOB_ and *bla*_CME_ genes from each *Elizabethkingia* clade were tested for MIC against 38 clinically relevant antimicrobials (Table 4). To date, *Elizabethkingia* species lack their own defined breakpoint, so they have been interpreted by using EUCAST non-species and NCSI non-Enterobacteriaceae PK-PD breakpoints (Supplementary data 12). All isolates tested showed remarkable resistance to carbapenems, cephalosporins, penicillins including carboxypenicillin and monobactam. Regarding different *bla*_GOB,_ *bla*_B_ and *bla*_CME_ combinations, some differences in resistance profiles were noted (Table 4), including piperacillin/tazobactam resistance in only *bla*_B-26-like_/*bla*_GOB-19-like_/*bla*_CME-variant_ *E. miricola* isolates, and cefepime resistance in two *E. anophelis* isolates (one *bla*_B-1-like_/*bla*_GOB-20_/*bla*_CME-1_ and one *bla*_B-1-like_/*bla*_GOB-variant_/*bla*_CME-1_).

**Table 4:** MIC data of aquatic environmental *Elizabethkingia* isolates from South Australia against clinically relevant antimicrobials. Cells colors: red = resistant, yellow = intermediate, green = sensitive. *bla*_B_, *bla*_GOB_ and *bla*_CME_ alleles shown under isolate names; *^V^* = variant *^L^* = like.

			*E. anophelis*			*E. umeracha* sp. nov.				*E. miricola*		
Antimicrobial	Range tested (µg/mL)	MIC 90 (µg/mL)	ER-QUAD-EK_14B-1^L^ GOB-20 CME-1	ER-QUAD-EK_56B-1^L^ GOB^V^CME-1	ER-QUAD-EK_18B-11 GOB20 CME-1	ER-QUAD-EK_08B^V^GOB^V^CME^V^	ER-QUAD-EK_09B^V^GOB^V^CME^V^	ER-QUAD-EK_10B^V^GOB^V^CME^V^	ER-QUAD-EK_21B^V^GOB^V^CME^V^	ER-QUAD-EK_94B-6^L^ GOB-25^L^CME^V^	ER-QUAD-EK_64B-26^L^ GOB-19^L^ CME^V^	ER-QUAD-EK_92B-26^L^ GOB19^L^ CME^V^
Amoxicillin	2–32	> 32	>32	>32	32	>32	>32	>32	>32	>32	>32	>32
Ampicillin	2–32	> 32	>32	>32	32	>32	>32	>32	>32	>32	>32	>32
Amoxicillin/clavulanic acid	4–128	> 128	8	8	16	8	16	16	8	8	16	16
Piperacillin/tazobactam	1–64	> 64	<1	<1	<1	<1	<1	2	<1	4	8	8
Ampicillin/sulbactam	8–128	> 128	32	16	16	32	32	64	32	32	32	32
Temocillin	2–32	> 32	> 32	16	> 32	> 32	> 32	> 32	> 32	> 32	> 32	> 32
Cephalexin	4–64	N/A	> 64	> 64	64	> 64	> 64	> 64	> 64	> 64	> 64	> 64
Cefazolin	0.25–32	> 32	> 32	> 32	> 32	> 32	> 32	> 32	> 32	32	> 32	> 32
Cefuroxime	1–16	> 16	>16	>16	16	>16	>16	>16	>16	>16	> 16	>16
Cefoxitin	8–256	N/A	< 8	< 8	16	32	32	32	32	16	32	32
Cefotaxime	0.03–8	> 8	> 8	>8	8	> 8	8	8	4	2	4	4
Ceftazidime	0.12–16	> 16	16	> 16	> 16	> 16	> 16	> 16	> 16	4	> 16	> 16
Ceftriaxone	0.03–4	> 4	> 4	> 4	> 4	> 4	> 4	> 4	> 4	2	> 4	> 4
Cefiderocol	0.03–32	N/A	8	4	8	4	4	4	1	1	4	8
Cefepime	0.06–16	> 16	>16	16	8	8	4	4	4	1	4	4
Ceftaroline	0.5–16	> 16	> 16	> 16	> 16	> 16	> 16	> 16	> 16	> 16	> 16	> 16
Ceftolozane/tazobactam	0.5–16	16	> 16	> 16	> 16	> 16	> 16	> 16	> 16	> 16	> 16	> 16
Meropenem	0.015–32	> 16	16	16	32	32	32	32	32	32	32	32
Tebipenem	0.03–8	N/A	8	4	8	8	8	8	4	> 8	8	8
Etrapenem	0.015–4	> 4	> 4	> 4	> 4	> 4	> 4	> 4	> 4	> 4	> 4	> 4
Aztreonam	0.5–16	> 16	> 16	> 16	> 16	> 16	> 16	> 16	> 16	> 16	> 16	> 16
Amikacin	1–64	64	32	4	32	32	32	32	32	16	16	16
Gentamicin	0.25–16	>16	8	2	> 16	16	8	8	8	8	4	2
Tobramycin	0.015–64	>16	>64	32	>64	> 64	16	64	64	64	64	64
Azithromycin	4–64	N/A	8	< 4	8	< 4	< 4	< 4	< 4	< 4	< 4	< 4
Ciprofloxacin	0.015–4	2	1	0.12	0.25	0.25	0.25	0.12	0.06	0.5	0.25	0.25
Levofloxacin	0.06–8	1	0.25	0.125	0.25	0.125	0.125	0.125	< 0.06	0.25	0.25	0.125
Trimethoprim	0.5–16	N/A	8	2	1	< 0.5	2	< 0.5	< 0.5	<0.5	2	2
Trimethoprim/sulfamethoxazole	0.12/2.38–32/608	2.38–152	8/152	1.0/19.0	0.5/9.5	> 0.12/2.38	0.5/9.5	0.25/4.75	<0.12/2.38	<0.12/2.38	0.5/9.5	0.5/9.5
Vancomycin	0.12–32	N/A	8	8	8	4	>32	4	4	8	8	8
Teicoplanin	2–64	N/A	> 64	> 64	> 64	> 64	> 64	> 64	> 64	> 64	> 64	> 64
Minocycline	0.25–16	1	0.5	< 0.125	0.25	0.5	0.5	0.5	< 0.125	0.5	0.25	0.5
Doxycycline	0.015–64	> 64	2	0.5	0.5	0.5	0.5	1	0.25	0.5	1	1
Tigecycline	0.12–8	2	4	2	8	4	4	4	8	4	2	4
Rifamicin	0.12–32	N/A	< 0.125	< 0.125	0.25	< 0.125	2	< 0.125	< 0.125	< 0.125	0.25	< 0.125
Colistin	0.25–8	N/A	> 8	> 8	> 8	> 8	> 8	> 8	> 8	> 8	> 8	> 8
Polymixin B	0.25–8	N/A	> 8	> 8	> 8	> 8	> 8	> 8	> 8	> 8	> 8	> 8
Chloramphenicol	2–128	>128	128	16	32	8	8	8	8	16	8	8

Isolates were also resistant to antibiotic classes other than carbapenem and ESBLs, including aminoglycosides and glycylcycline (Table 4). One isolate, *E. anophelis* ER-QUAD-EK_14 was highly resistant to chloramphenicol and trimethoprim/sulfamethoxazole. Azithromycin and rifampicin have no corresponding breakpoint in EUCAST or CLSI, however, tested isolates had a very low MIC (up to the lowest range tested), suggesting a potential sensitive profile. Vancomycin and teicoplanin also lack a breakpoint but their MICs were very high, indicating non-susceptibility. Likewise for the glycopeptides and colistin, which showed higher MIC than the top of concentration tested, suggesting potential resistance of *Elizabethkingia* against these antibiotics.

### 3.9 Mobile genetic elements characterization: ICEs, plasmids and phages

Integrative conjugative elements were identified in 67 / 94 (71.3%) of the aquatic environments *Elizabethkingia* spp. by comparing to *Elizabethkingia* ICE sequences publicly available in Genbank, comprising three types of ICEs (Xu et al., 2019): ICEEaI from strain CSID3015183678, ICEEaII from strain NUHP1 and ICEEaIII from strain R26. The alignments demonstrated imperfect matches to the reference sequences, however all matches were closest to the type III ICE from *E. anophelis* strain R26.

Two plasmids have been described from *Elizabethkingia* species so far (Accessions CP016375.1 and CM003640.1), however neither were detected in this study. In our analysis, both plasmid sequences were aligned to the aquatic *Elizabethkingia* isolates from this collection, as well clinical *Elizabethkingia* isolates from Australia sourced from Genbank. Thirteen (81%) of the environmental *E. anophelis* in clade 2 showed low-quality matches to plasmid CP016375.1 (average 8% coverage at 90% identity) (Supplementary Data 13). Alignments to the second reference plasmid from *E. miricola* strain EM_CHUV (CM003640.1) revealed little to no homology.

We performed a phage analysis on 94 *Elizabethkingia* genomes via Phaster, with hits detected for each isolate (data not shown). While most hits were recorded as 'incomplete' due to scaffolding of the WGS, fourteen isolates were detected with complete phages (Supplementary Data 14). From these fourteen isolates, coming from multiple species, we selected four ranges of hit length to group the phages: 48.7 kb, 21.4–27.1 kb, 10.4 −18.8 kb, and 4.9–9.7 kb. Alignments were generated to identify similar phages amongst the different size ranges, with only the largest (48.7 kb) appearing conserved in multiple isolates. Initially, this phage was detected in two *E. miricola*, one from a wetland and another from a river, with alignments demonstrating different chromosomal locations. By aligning the large phage against the Australian *Elizabethkingia* sequences, we identified 17 environment and clinical *Elizabethkingia* isolates to carry it: two *E. anophelis* (one from a wetland and another from a human bronchial alveolar lavage), nine *E. miricola* (seven from wetlands and two from a river), one *E. bruuniana* (human blood) and five *E. umeracha* sp. nov. (dam). While short-read assembly data prevents detailed comparisons of these phages, it is clear the *Elizabethkingia* isolates share mobile DNA.

## 4 Discussion

*Elizabethkingia* spp. are emerging pathogens and the only known organisms with multiple chromosomal metallo-β-lactamase genes, offering inherent resistance to carbapenems (Hu et al., 2020). *Elizabethkingia* species are considered environmental bacteria, with water bodies serving as environmental reservoirs. Contaminated water is implicated in *Elizabethkingia* spp. transmission pathways (Booth, 2014), yet with the exception of insects (Kämpfer et al., 2011), frogs (Hu et al., 2017; Lei et al., 2019), reptiles (Jiang et al., 2017) and spacecraft (Li et al., 2003), most studies on *Elizabethkingia* genus have focused on clinical isolates and isolates taken from hospital environments, leaving *Elizabethkingia* species dwelling in aquatic environments unexplored. Here we characterized WGS of 94 *Elizabethkingia* derived from dam, river, and wetland samples from South Australia, thereby providing the first study of *Elizabethkingia* from diverse aquatic environments. Furthermore, we provide comparative genomics analyses of these environmental isolates with clinical *Elizabethkingia* isolates originating from Australia and worldwide.

Correctly identifying *Elizabethkingia* species is paramount as not only is the literature on *Elizabethkingia* spp. convoluted due to various nomenclature changes, but standard commercial microbial identification systems, such as biochemical tests and mass spectrometry (MS) using standard databases, cannot currently differentiate *E. anophelis, E. bruuniana, E. ursingii* or *E. occulta* and these are often misidentified as either *E. meningoseptica* or *E. miricola* (Chen et al., 2019; Burnard et al., 2020; Lin et al., 2019b; Snesrud et al., 2019). Consistently, our initial MALDI-TOF MS results misidentified 11 isolates as *E. meningoseptica*. Interestingly, all speciation methods we used, including MALDI-TOF MS, Kraken2 and SpeciesFinder, gave conflicting results. The issues regarding MS and Kraken2 misidentifications likely arose from using standard databases (Lin et al., 2019a) while SpeciesFinder uses 16S rRNA gene sequences, which are known to be limited for taxonomic purposes (Larsen et al., 2014) with studies demonstrating less than 30% accuracy for aerobic bacteria to the species level (Teng et al., 2011). These data suggest that *in lieu* of WGS, future *Elizabethkingia* spp. studies should be cautious in using 16S rRNA for speciation and ensure any utilized MS databases include all *Elizabethkingia* species.

The fact that *E. anophelis* was misidentified here and elsewhere (Chen et al., 2019; Han et al., 2017; McTaggart et al., 2019; Snesrud et al., 2019) as *E. meningosepticum* by conventional clinical methods has led to speculation that *E. anophelis* is not only underrepresented but may actually be the primary species to cause disease in humans. This hypothesis is strengthened by recent reports of life-threatening *E. anophelis* infections in Asia, Australia, and the USA (Burnard et al., 2020; Lin et al., 2019a). The 16 *E. anophelis* isolates identified here in dam and wetland samples formed two clades which differed by approximately 807 SNPs. The single wetland isolate and two dam isolates were found to differ by only ∼36 SNPs to three *E. anophelis* isolates originating from sepsis patients in Queensland, Australia, and ∼42 SNPs to two *E. anophelis* isolates derived from sinks located in a Queensland hospital (Burnard et al., 2020). Screening for putative virulence factors did not identify any specific to the three environmental isolates and the three sepsis isolates, however several putative virulence factors were found to be unique to *E. anophelis* in general, including homologs of lipopolysaccharide biosynthesis proteins and serum resistance protein OmpA.

The majority of Australian aquatic environment isolates from this study were *E. miricola. E. miricola* is known to cause sepsis, oral and urinary tract infections (Green et al., 2008; Lin et al., 2019a; Zdziarski et al., 2017) though reports of infection are less frequent than that for *E. meningoseptica* and *E. anophelis*. Environmental *E. miricola* from this study formed two distinct clades. While a clade of ten isolates were phylogenetically positioned next to three Australian clinical *E. miricola* isolates taken from sputum samples (Burnard et al., 2020), the average SNP difference between these environmental and clinical isolates was 21,539. Nevertheless, environmental *E. miricola* shared putative virulence factors with clinical strains including homologs of capsule protein, Cps4I (Mirza et al., 2018), haemolytic toxin SmcL (González-Zorn et al., 2000), and iron acquisition protein YbtP (Fetherston et al., 1999). The remaining 61 *E. miricola* isolates formed a clonal clade with an average difference of 7 SNPs. Despite the highly clonal nature of these isolates, they originated from two wetland sites approximately 10 km apart. Identifying potential transmission pathways, such as through wildlife, will be critical for future epidemiology.

The remaining seven *Elizabethkingia* isolates originated from dam samples and were phylogenetically placed proximal to *E. bruuniana* strains, though SNP analyses demonstrated the two branches to differ by ∼124,216 SNPs. This considerable difference prompted an investigation as to whether these seven isolates represent a novel *Elizabethkingia* species. WGS-based ANI and *in silico* DDH analyses are known to be robust speciation methods that have proven more accurate than even the gold-standard conventional DDH for bacterial species delineation (Konstantinidis and Tiedje, 2005; Lin et al., 2019a; Meier-Kolthoff et al., 2013; Richter and Rosselló-Móra, 2009; Varghese et al., 2015). Here, both analyses clearly indicated that these isolates were a distinct species. The *rpoB* gene is also used in speciation and possesses a higher resolution for delineation than the 16S rRNA gene (Adékambi et al., 2009). Here, all seven isolates fell under the 97.7% similarity cut-off to be classified as *E. bruuniana*. Furthermore, we identified 1886 genes unique to these isolates and where functional assignment was possible, these genes were mainly involved in cellular and metabolic processes. Together these data indicate a closely related but distinct species to *E. bruuniana* and we propose the name *E. umeracha* sp. nov. Though these isolates were shown here to share 62 putative virulence factors with *E. anophelis* and *E. miricola* isolates, future studies are required to determine whether this new species is pathogenic.

Metallo-β-lactamases (MBLs) are a worldwide concern as they confer resistance against carbapenems and almost all β-lactams (Chang et al., 2019), making pathogens carrying MBLs very difficult to treat. *Elizabethkingia* are currently the only known organisms to carry two chromosomal MBLs (*bla*_B_ and *bla*_GOB_) and additionally carry a chromosomal *bla*_CME_ gene conferring resistance to cephalosporins (González and Vila, 2012). Here we found that the three species of *Elizabethkingia* residing in aquatic environments all carried multiple known alleles, as well as novel variants of *bla*_B_, *bla*_GOB_ and *bla*_CME_ genes. Regardless of allelic combinations, MIC testing demonstrated all were highly resistant to carbapenems, penicillins and monobactams. All tested isolates were also resistant to cephalosporins, though one *E. miricola* isolate carrying a *bla*_CME_ variant was susceptible to cefepime. Intravenous vancomycin has been cited as the favorable treatment option for *Elizabethkingia* infections (Jean et al., 2017). While most tested isolates had MICs of vancomycin at 4 or 8 µg/mL, one *E. umeracha* sp. nov. isolate had > 32 µg/mL, suggesting that a conventional dose of vancomycin would not be effective (Chang et al., 2019). In addition to carbapenems and β-lactams, several isolates were found resistant to aminoglycosides and one *E. anophelis* isolate was highly resistant to trimethoprim/sulfamethoxazole and chloramphenicol. However, no additional AMR genes were identified, suggesting the presence of novel AMR mechanisms.

Environmental bacteria often harbor important AMR genes that are later captured and disseminated by more common human pathogens. For example, the ESBL gene, *bla*_CTX__−__M_, now endemic among *Enterobacteriaceae*, likely originated from *Kluyvera ascorbate*, a soil bacteria (Humeniuk et al., 2002). The acquisition and spread of resistance, as well as virulence genes, is facilitated by MGEs. ICEs are MGEs that integrate into a host chromosome and can bestow new phenotypes (Xu et al., 2019). In the 2015–2016 *E. anophelis* outbreak in the USA, which led to 66 confirmed cases of sepsis and 19 deaths, an ICE was identified in all of the outbreak clones. This ICE interrupted the *mutY* gene which led to a hypermutator phenotype (Perrin et al., 2017). ICEs were identified in 71% of 94 environmental *Elizabethkingia* isolates, however these bore no similarity to the ICE associated with the USA outbreak and only ∼50% similarity to the type III ICE found in *E. anophelis* strain R26 (Kämpfer et al., 2011), the first *E. anophelis* strain isolated. As such we have extended knowledge on the types of ICE that are found in *Elizabethkingia* spp..

In conclusion, we presented the first WGS analysis of *Elizabethkingia* species found in aquatic environments and discovered that they carry diverse *bla*_B_ *bla*_GOB_ and *bla*_CME_ genes and are highly resistant to carbapenems, cephalosporins, monobactams and other beta-lactams. Some isolates were also resistant to additional antibiotic classes suggesting the presence of yet undiscovered AMR mechanisms. We uncovered environmental *E. anophelis* isolates that were very closely related to sepsis-causing clinical strains, thus identifying water bodies as an important reservoir for pathogenic *Elizabethkingia* spp. and highlighting the potential for cross-habitat movement. Finally, we discovered a proposed novel species, *E. umeracha* sp. nov., representatives of which appear resistant to vancomycin and carry novel metallo β-lactamase and extended-spectrum serine β-lactamase gene alelles.

## CRediT authorship contribution statement

**Sopheak Hem:** Writing – original draft, Formal analysis, Investigation, Writing – review & editing. **Veronica M. Jarocki:** Writing – original draft, Formal analysis, Investigation, Supervision, Writing – review & editing. **Dave J. Baker:** Investigation, Resources. **Ian G. Charles:** Investigation, Resources. **Barbara Drigo:** Investigation, Conceptualization, Validation, Formal analysis, Data curation, Writing – review & editing. **Sarah Aucote:** Investigation, Formal analysis, Data curation. **Erica Donner:** Conceptualization, Funding acquisition, Supervision, Writing – review & editing. **Delaney Burnard:** Resources, Methodology. **Michelle J. Bauer:** Resources, Methodology. **Patrick N.A. Harris:** Resources, Supervision, Writing – review & editing. **Ethan R. Wyrsch:** Investigation, Methodology, Validation, Writing – review & editing. **Steven P. Djordjevic:** Resources, Supervision, Funding acquisition, Project administration, Writing – review & editing.

## CRediT authorship contribution statement

**Sopheak Hem:** Writing – original draft, Formal analysis, Investigation, Writing – review & editing. **Veronica M. Jarocki:** Writing – original draft, Formal analysis, Investigation, Supervision, Writing – review & editing. **Dave J. Baker:** Investigation, Resources. **Ian G. Charles:** Investigation, Resources. **Barbara Drigo:** Investigation, Conceptualization, Validation, Formal analysis, Data curation, Writing – review & editing. **Sarah Aucote:** Investigation, Formal analysis, Data curation. **Erica Donner:** Conceptualization, Funding acquisition, Supervision, Writing – review & editing. **Delaney Burnard:** Resources, Methodology. **Michelle J. Bauer:** Resources, Methodology. **Patrick N.A. Harris:** Resources, Supervision, Writing – review & editing. **Ethan R. Wyrsch:** Investigation, Methodology, Validation, Writing – review & editing. **Steven P. Djordjevic:** Resources, Supervision, Funding acquisition, Project administration, Writing – review & editing.

## Declaration of Competing Interest

The authors declare that they have no known competing financial interests or personal relationships that could have appeared to influence the work reported in this paper.

## Appendix Supplementary materials

Image, application 1

Image, application 2

Image, application 3

Image, application 4

Image, application 5

Image, application 6

Image, application 7

Image, application 8

Image, application 9

Image, application 10

Image, application 11

Image, application 12

Image, application 13

Image, application 14
