# Supplementary material for: Genomic analysis of Elizabethkingia species from aquatic environments: Evidence for potential clinical transmission
Source: Curr Res Microb Sci. 2021 Nov 26;3:100083. doi: 10.1016/j.crmicr.2021.100083 (PMC8703026; doi:10.1016/j.crmicr.2021.100083)
Supplement: Supplementary file 3 [file mmc3.pdf]

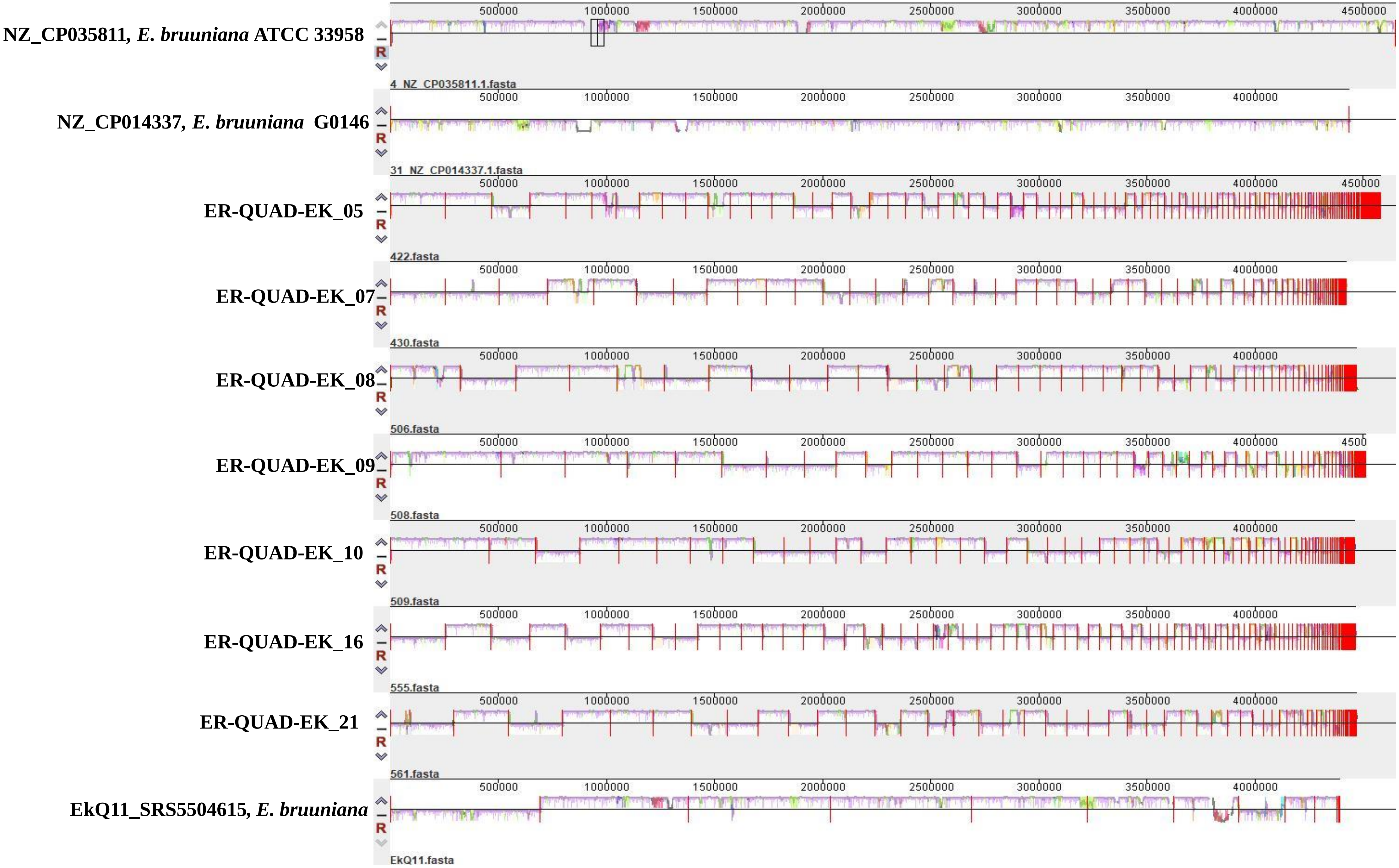
NZ_CP035811, E. bruuniana ATCC 33958
4_NZ_CP035811.1.fasta
NZ_CP014337, E. bruuniana G0146
31_NZ_CP014337.1.fasta
ER-QUAD-EK_05
422.fasta
ER-QUAD-EK_07
430.fasta
ER-QUAD-EK_08
506.fasta
ER-QUAD-EK_09
508.fasta
ER-QUAD-EK_10
509.fasta
ER-QUAD-EK_16
555.fasta
ER-QUAD-EK_21
561.fasta
EkQ11_SRS5504615, E. bruuniana
EkQ11.fasta
500000
1000000
1500000
2000000
2500000
3000000
3500000
4000000
4500000
