# Supplementary material for: Genomic analysis of Elizabethkingia species from aquatic environments: Evidence for potential clinical transmission
Source: Curr Res Microb Sci. 2021 Nov 26;3:100083. doi: 10.1016/j.crmicr.2021.100083 (PMC8703026; doi:10.1016/j.crmicr.2021.100083)
Supplement: Supplementary file 10 [file mmc10.pdf]

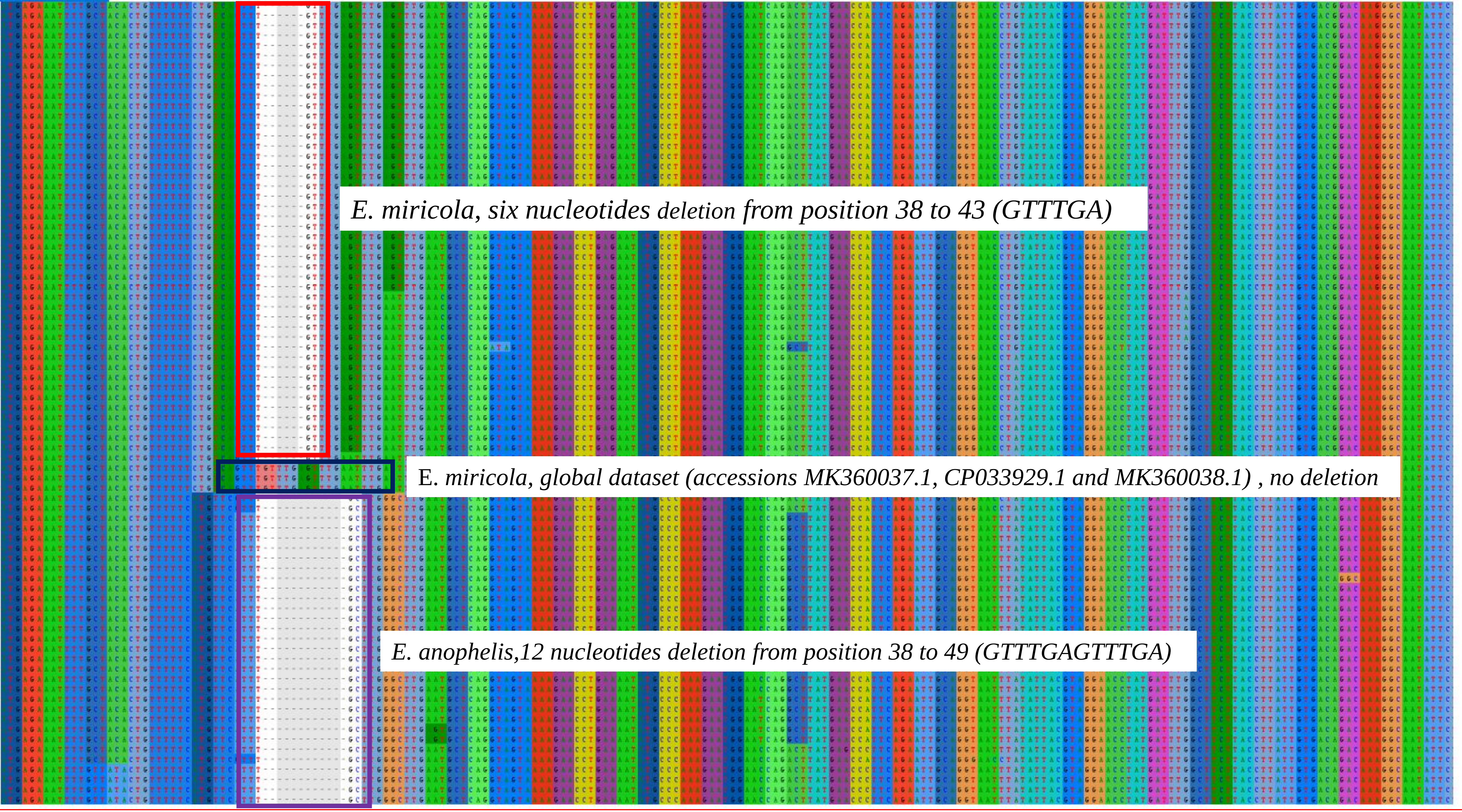
E. miricola, six nucleotides deletion from position 38 to 43 (GTTTGA)
E. miricola, global dataset (accessions MK360037.1, CP033929.1 and MK360038.1) , no deletion
E. anophelis,12 nucleotides deletion from position 38 to 49 (GTTTGAGTTTGA)
