# Supplementary figures and images for: Genomic analysis of Elizabethkingia species from aquatic environments: Evidence for potential clinical transmission
Source: Curr Res Microb Sci. 2021 Nov 26;3:100083. doi: 10.1016/j.crmicr.2021.100083 (PMC8703026; doi:10.1016/j.crmicr.2021.100083)

Tree scale: 0.01

# *bla<sub>CME</sub>* Tree

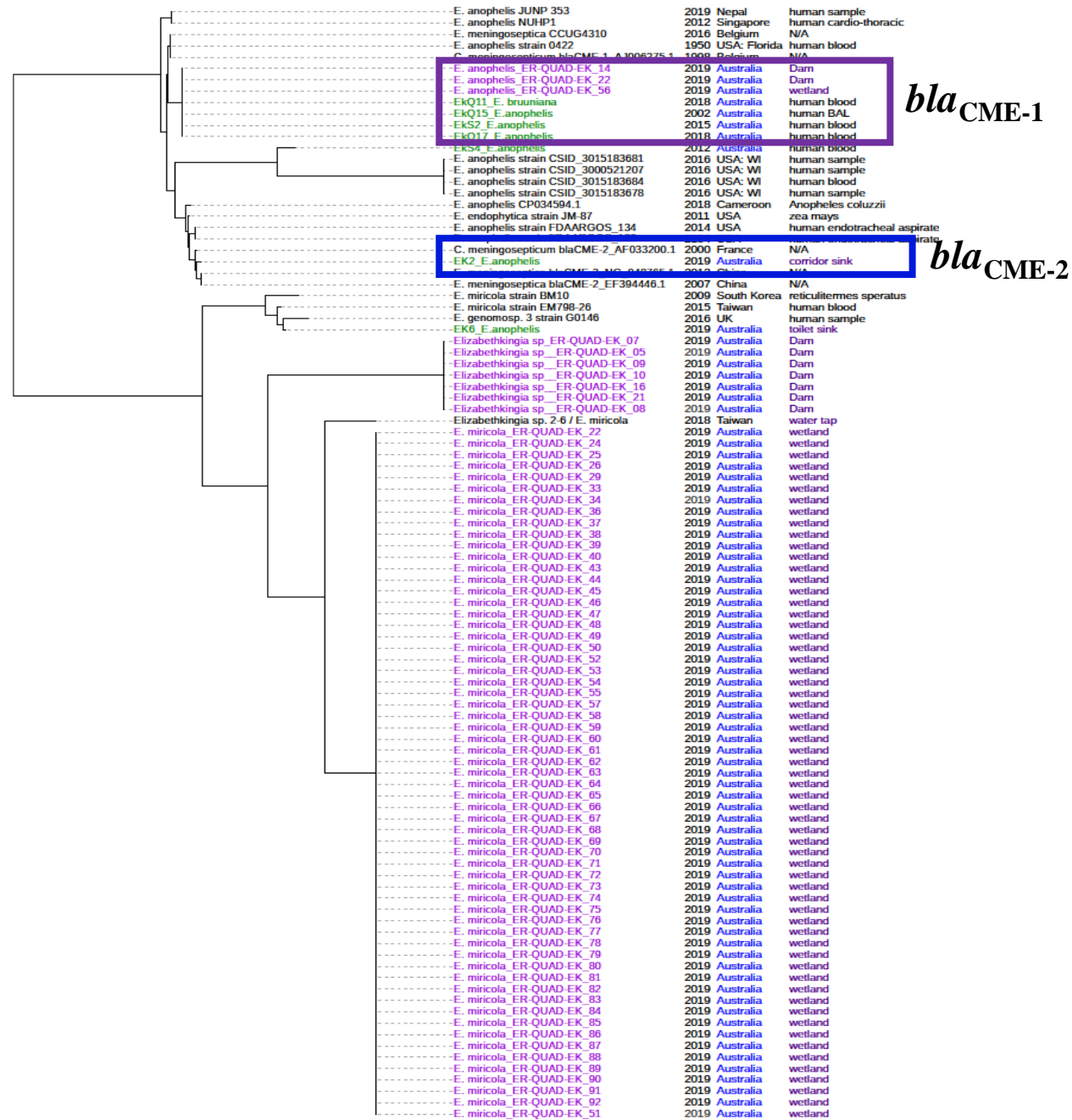

Supplement: Supplementary file 11 [file mmc11.pdf]
