# Supplementary material for: Genomic analysis of Elizabethkingia species from aquatic environments: Evidence for potential clinical transmission
Source: Curr Res Microb Sci. 2021 Nov 26;3:100083. doi: 10.1016/j.crmicr.2021.100083 (PMC8703026; doi:10.1016/j.crmicr.2021.100083)
Supplement: Supplementary file 13 [file mmc13.pdf]

Plasmid alignments of all *E. anophelis* from aquatic environment (n=16) and clinical isolates (n=22) to reference plasmid CP016375.using BRIG

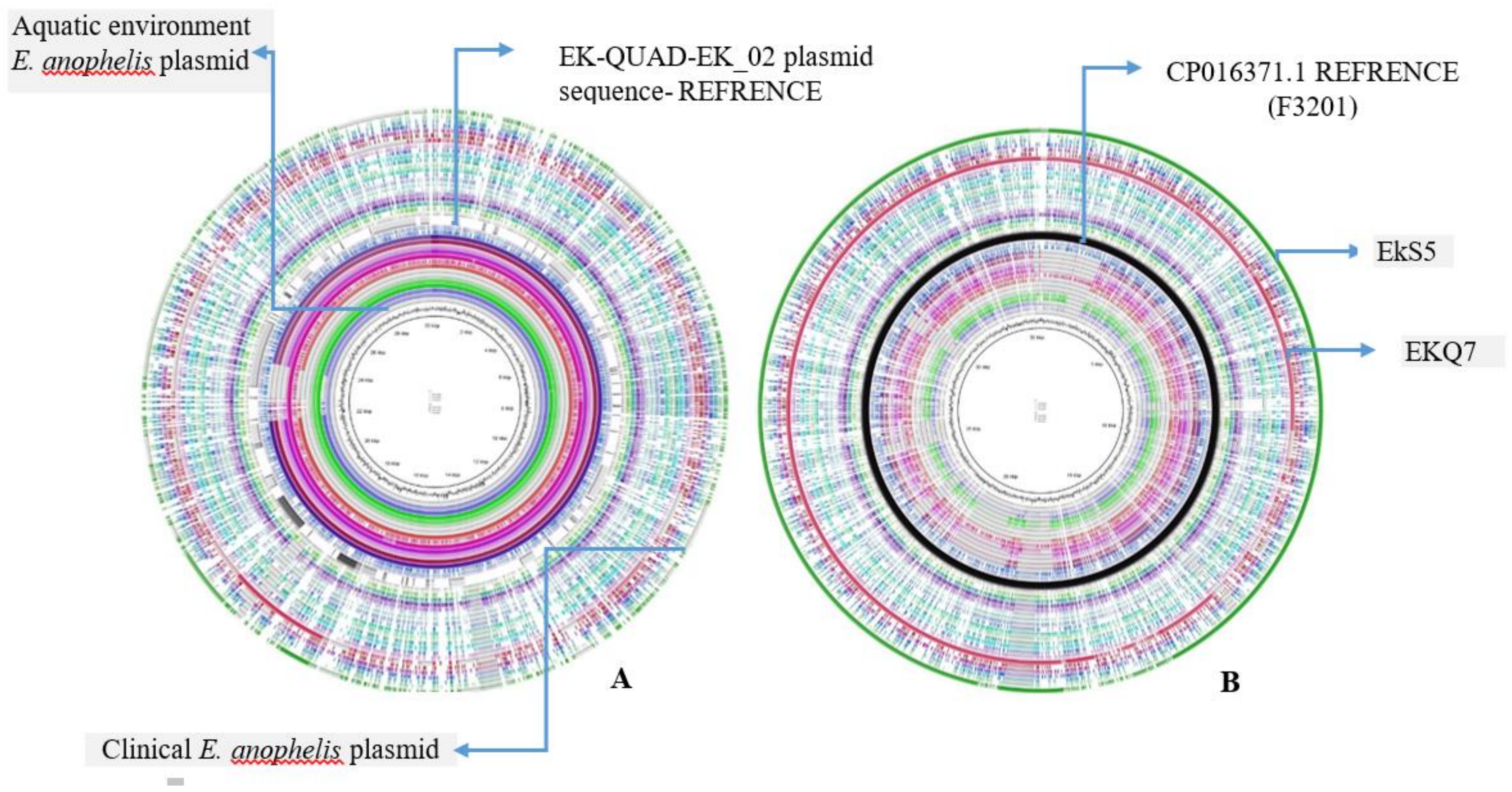

(a) shows plasmid maps and alignment of a representative plasmid from *E. anophelis* from Australian aquatic environment (EK-QUAD-EK_02) against all plasmids identified from *E. anophelis* in this study (aquatic environment and clinical isolates). Inner rings show Blastn alignment against related plasmid sequences (from inside to outside started by aquatic environment isolates followed in order by clinical isolates). Center histogram shows GC content.

(b) An analysis but using a plasmid reference from Genbank with accession CP016375 from strain F3201.
